# The augmentative role of chitosan and platelet-rich fibrin in reducing epidural fibrosis after laminectomy in rats

**DOI:** 10.1186/s40001-025-02951-3

**Published:** 2025-08-14

**Authors:** Samah Fouad, Marwa Abass, Awad Rizk, Esam Mosbah, Mostafa M. Nabeeh, Ayman S. Elmezayyen, M. I. El-Henawey, Adel Zaghloul

**Affiliations:** 1 Medical Experimental Research Center (MERC), Faculty of Medicine, Mansoura University, Mansoura, 35516 Egypt; 2 Department of Surgery, Anesthesiology and Radiology, Faculty of Veterinary Medicine, Mansoura University, Mansoura, 35516 Egypt; 3 Department of Neurosurgery, Faculty of Medicine, Mansoura University, Mansoura, 35516 Egypt; 4 Physics Department, Faculty of Science, Mansoura University, Mansoura, 35516 Egypt; 5 Physics Department, Faculty of Science, New Mansoura University, New Mansoura, 35516 Egypt

**Keywords:** Chitosan, Platelets-rich fibrin, Laminectomy, Epidural fibrosis

## Abstract

**Background:**

Epidural fibrosis (EF) is the major complication that develops in the operative region of the spinal vertebrae. This fibrous scar connects the connective tissue around the lateral nerve roots and epidural, resulting in severe pain post-spinal operation and impairment of the nerves’ function.

**Purpose:**

This study was conducted to investigate the effect of using platelet-rich fibrin (PRF), chitosan, and their combination in reducing epidural fibrosis after laminectomy in rats.

**Methods:**

Ninety male Sprague Dawley rats weighing 255 ± 55 g were randomly assigned to five groups, each consisting of 15 rats: the normal group (control), the laminectomy group, the PRF group, the chitosan group, the combination group (PRF/chitosan), and the donor group. All rats, except the control group, had lumbar laminectomy surgeries between L3 and L5. Macroscopic analysis, histological evaluations, and mRNA analysis for TGFβ-1 and IL6 were compared statistically after a 30-day follow-up.

**Results:**

In comparison to the laminectomy group, the EF area was significantly decreased in the PRF, chitosan, and combination groups. Histological study, macroscopic inspection, and mRNA expression of TGFβ-1 (*P* < 0.0001) and IL6 (*P* < 0.0001) show that the use of PRF with chitosan topically in dura following laminectomy resulted in a decrease in scar tissue formation, inflammation, and EF post-laminectomy.

**Conclusion:**

The combination of chitosan with PRF is a potential therapeutic approach for minimizing EF in rats after laminectomy.

**Graphical Abstract:**

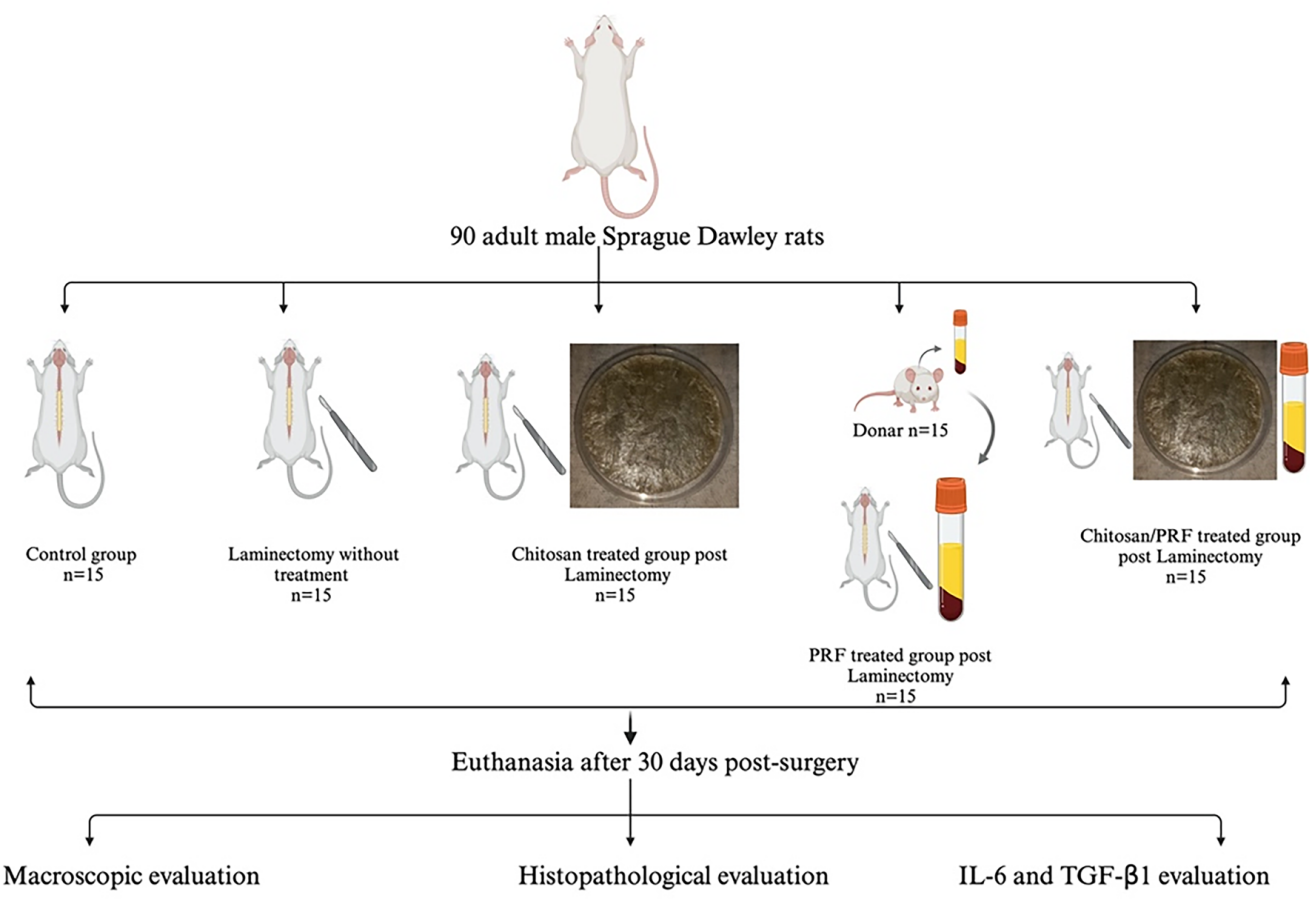

## Introduction

Laminectomy is the most common surgical procedure used in the treatment of different spinal problems, such as lumbar spinal stenosis [1]. One of the main reasons for failed back surgery syndrome (FBSS), which is characterized by persistent pain following surgery, is EF, which arises from the overabundance of healing tissue in this region and results in adhesions in the dura mater and nerve roots [2]. FBSS is used to describe lower back and/or lower extremity pain that is new, persistent, or recurring after one or more spine procedures [3]. In addition to creating aggravation for patients with neuropathic pain, who endure increased discomfort and reduced physical functioning and quality of life, this presents an increasing challenge for medical practitioners treating spinal illnesses [4].

It has been challenging lately to identify the underlying source of back pain following lumbar spine operations. The most common ones include surgeries on EF, spinal instability, root degeneration, recurrent disc herniation, spinal stenosis, foreign body reaction, pseudoarthrosis, and improper spinal level causes [5].

Epidural fibrosis EF is the growth of connective scar tissue at the surgical site following spinal surgeries. The epidural and/or lateral nerve roots are surrounded and adhered to by this scar tissue, which results in pain following spine surgery and functional impairment [6]. Numerous clinical and experimental studies on medications that aim to prevent or lessen EF following laminectomy have been conducted [7–12]. In addition, the following substances were assessed: cinnamon aldehyde [13], medical ozone treatment [14], Tubastatin A, Laminin α1, Tranexamic acid [7], berberine [15], laminin α1 [16], tubastatin A [17], and metformin [18].

PRF is an immunological and platelet concentration that includes every element of a blood sample that aids in fostering healing and immunity. Post-laminectomy, PRF lowers the density of chronic inflammatory cells in rats and, via platelet-generated cytokines and growth factors, stimulates tissue healing, angiogenesis, and osteoblast proliferation [19].

Transdermal medication delivery and tissue engineering have made extensive use of chitosan (Chi), a renewable, useful, nontoxic, and biodegradable biopolymer [20]. According to the report, it has been found that using Chi with triple compression bandages improves venous leg ulcers [21]. The Chi barrier effectively reduces peridural adhesions and EF in a rabbit model following the laminectomy [22]. The article states that venous leg ulcers have been proven to improve when Chi is used in conjunction with triple compression bandages [23].

A combination of PRF with chitosan is utilized in wound dressings to promote faster wound healing, regulate the hydrogel dressing’s protein release, speed up re-epithelialization, and lower the risk of infection in the wound area [24]. The presence of the chitosan membrane resulted in a well-organized tissue connected with the surrounding vertebral bone tissue, with evidence of regeneration of bone tissue in continuity with the native bone [25]. The inclusion of PRF and chitosan will prevent epidural fibrosis by affecting the membrane’s mechanical characteristics, cell survival, and rate of degradation [26].

## Methods

### Animals

A cohort of 90 mature male Sprague Dawley rats weighing 255 ± 55 g was used in this study. This work's experimental protocol was authorized by the National Research Center-Egypt’s Ethics Committee through the Faculty of Veterinary Medicine at Mansoura University. It was registered under the Ph.D./102 registration number. The animals were kept as comfortable as possible during the procedure and killing.

The rats were randomized into five groups (15 per group). The groups were categorized as follows: normal, which was a control negative group. The laminectomy group consisted of rats that underwent laminectomy. The PRF-treated group consisted of rats that received PRF on the dura mater following laminectomy. The chitosan-treated group consisted of rats that received chitosan on the dura mater following laminectomy. The PRF and chitosan combination consisted of rats that received PRF and chitosan on the dura mater following laminectomy. The other 15 donor rats were used to collect the blood samples to prepare PRF.

### Preparation of the PRF clot

**Fig. 1:**
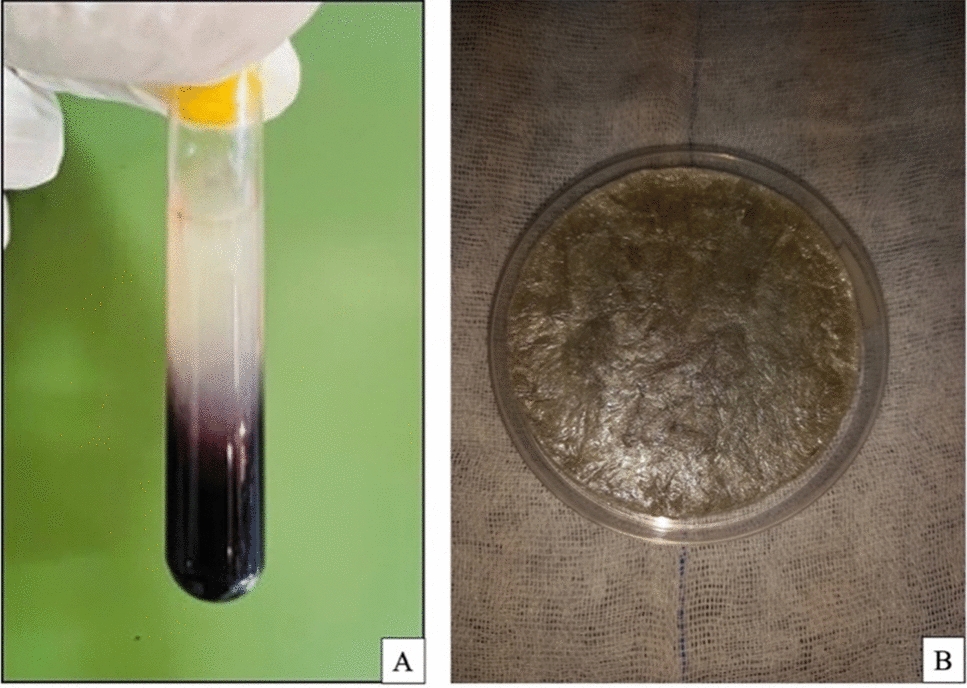
A Platelet-rich fibrin (PRF) clot. B Chitosan film

According to [27], 5 ml of the blood samples were obtained from 15 donor rats before laminectomy through cardiac puncture. The blood sample was collected into a glass centrifuge tube (Shanghai Goldenwell Medical Technology Co, Ltd., China) without the presence of an anticoagulant. Subsequently, the tubes were promptly subjected to centrifugation for 10 min at a speed of 3000 revolutions per minute (rpm) (Hettich EBA 8S centrifuge, D-78532 Tuttlingen, Germany) (RCF = 402 × g) at a 45^0^-rotor angulation with a radius of 40mm, resulting in the formation of three centrifugation strata: a fibrin clot (PRF) in the center of the tube, situated between the acellular plasma at the top and the red corpuscles at the bottom. A considerable number of platelets were trapped in the thick fibrin strands. The upper layer, with a straw-colored appearance, was removed, and the intermediate fraction was extracted from the clot using a long-lasting, self-generated fibrin membrane (Fig. 1A).

### Preparation of chitosan solution

Chitosan solution was made by using a magnetic stirrer set at 500 rpm for 2 h to dissolve 1 gm of chitosan powder (> 85% Deacetylation degree, Meron, India) in 50 ml of deionized water with 1.25 ml of acetic acid 2.5% at room temperature 25 °C. Acetic acid 2.5% used to protonate the amine groups on the chitosan molecule, making it soluble in water and acidity pH increasing the effectivity in inhibiting bacterial growth [28].

The chitosan solution was sealed with cling film and left overnight to remove air bubbles. After getting a clear solution, the chitosan film was formed by casting it into a petri plate and letting it dry for 3 days at room temperature 25 °C (Fig. 1B).

### Scanning electron microscopy (SEM)

**Fig. 2:**
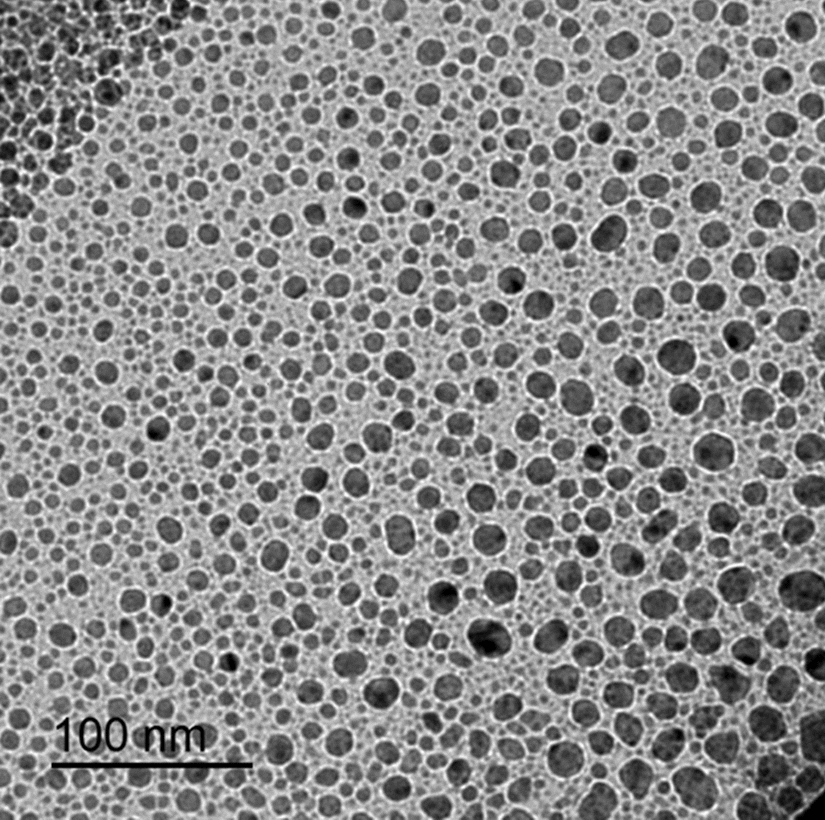
SEM images of different sizes of chitosan film at 100 nm magnifications

The surface of the chitosan film microstructures and cross-sectional distribution were analyzed using SEM in a JEOL 6360LV microscope (JEOL Inc., Peabody, MA, USA) in high vacuum mode, operating at 20 kV and a spot size 11. Scanning was done at Faculty of Agriculture, Electron microscope unit, Mansoura University (Fig. 2).

### Study design

Animals were administered an intramuscular antibiotic injection (Ampicillin; 15 mg/200 gm BW). In addition, Ophthalmic Ointment (Terramycin; Pfizer; Egypt) was applied to the eyes to prevent drying, and a heating electric blanket was used to keep the animals' body temperatures at 37 °C. Immediately before surgery, the surgical site (back of the animal) was aseptically prepared.

**Fig. 3:**
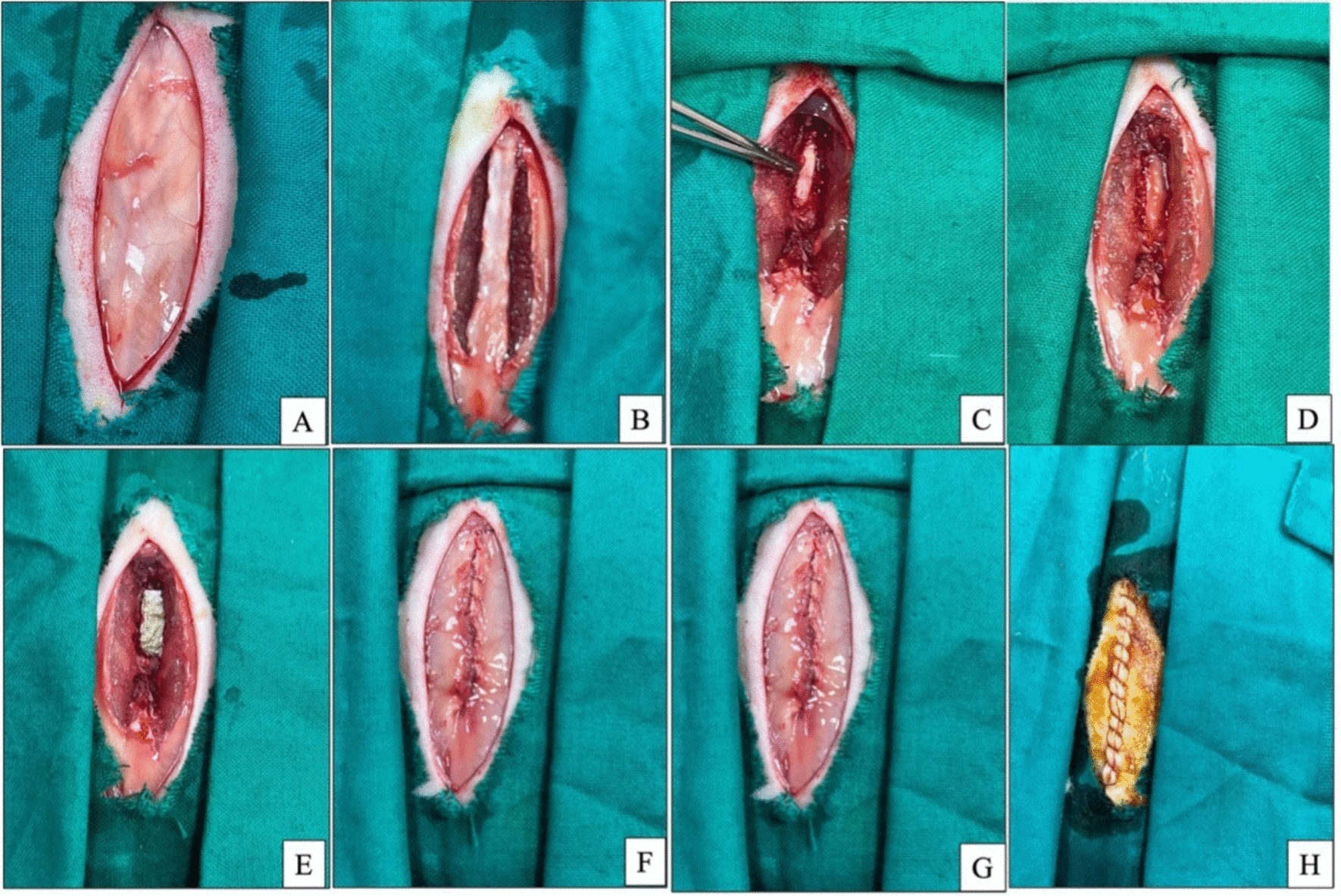
Laminectomy process in rats. **A** Skin incision at the dorsal spinous processes of the L3 and L5 levels. **B** Dissection of muscle and subcutaneous tissue. **C** incision of ligamentum flavum. **D** Total laminectomy at the L3 vertebral level. **E** Chitosan/PRF application on the surgical site. **F** muscle suture. **G** Subcutaneous suture. **H** Skin suture

Rats were anesthetized generally by intraperitoneal administration of 10 mg/kg Xylazine HCl (Xylajet 20 mg/ml, ADWIA, Egypt) and 75 mg/kg ketamine HCl (Ketamax 50 mg/ml, Troikaa Pharmaceuticals Ltd, Gujarat, India). After the rats were placed in a ventral position, their lower backs were sterilely prepared. A longitudinal skin incision was made in the midline between the dorsal spinous processes of the L3 and L5. The lumbosacral fascia was incised longitudinally, and the paraspinal muscles on both sides were dissected subperiosteally to reveal the laminae in the L3–L5 region. The ligamentum flavum and epidural adipose tissue were removed from the surgical site following a total laminectomy at the L3 vertebral level. The dura mater remained intact and exposed. Hemostasis was performed using cotton pads (Fig. 3).

### Euthanasia and sample collection

Animals were killed by intraperitoneal administration of 120 mg/kg of thiopental sodium (500 gm; E.I.P.C.O; Egypt) after 30 days post-laminectomy. Randomly selected rats were humanely euthanized, followed by intracardial perfusion with 4% paraformaldehyde solution. Ten rats were selected from each group for histopathological examination, while the remaining five rats from each group were used for macroscopic examination of their surgical sites.

After being killed, for 5 days, the en bloc spinal columns between L3 and L5 were collected and immersed in a 10% neutral-buffered formaldehyde solution. After 3 weeks of decalcification in 10% EDTA (Sigma Aldrich, St. Louis, MO, USA) [29], the specimens were embedded in paraffin.

En bloc vertebral columns between L3 and L5 were removed after killing and placed in 10% neutral-buffered formaldehyde solution for 5 days. After 3 weeks of decalcification in 10% EDTA (Sigma Aldrich, St. Louis, MO, USA), the specimens were embedded in paraffin. The region was divided into three sections: distal, middle, and proximal. These sections were then placed in sample cassettes. Furthermore, H&E staining was used to stain axial slices of the laminectomy site measuring 5 μm. A light microscope was utilized to evaluate epidural scar adhesion.

### Assessment of epidural fibrosis

#### Macroscopic assessment

The Rydell classification [30] was used to evaluate the EF degree at the laminectomy region: grade 0 indicates that the epidermis did not adhere to the dura mater, while, grade 1 means that the epidermis was adherent to the dura mater but was easily dissectible, grade 2 means that the epidermis was adherent to the dura mater and was difficult to remove without injury of the dura mater, and grade 3 indicates that the epidermis was firmly adherent to the dura mater and could not be dissected.

#### Histological analysis

He et al. [12] was used to determine the EF grades as the following: Grade 0—the dura mater was free from scar tissue covering and Grade 1—the dura mater was covered with a thin fibrous band and scar tissue; Grade 2—the dura referred was covered with scar tissue less than two-thirds of the laminectomy defect. Grade 3 refers to scar tissue that covers more than two-thirds of the laminectomy defect, spreading to nerve roots and arachnoids.

#### Quantitative morphometric analysis

A quantitative morphometric analysis was performed on the sections using Image J, 1.46a, NIH, USA, an image analysis program. A 400X magnification was used to quantify the dura matter thickness (at three separate sites). Mean values were used for statistical analysis. EF was then graded using a technique that was developed [12] as mentioned in Table 1. Additionally, arachnoid involvement in the scar tissue was recorded.

**Table 1:** The score of the scar tissue formation, inflammation, and density of scar tissue

Grade	Indication
Scar tissue formation score	
0	Scar tissue in less than two-thirds of the laminectomy defect
1	Scar tissue on the dura matter
2	Thin fibrous band separates the dura mater, and scar tissue
3	Scar tissue extending to the nerve roots or covering more than two-thirds of the laminectomy defect
Inflammation score	
0	Absent
1	Mild
2	Moderate
3	Severe
The density of scar tissue score	
0	Absent
1	Mild
2	Moderate
3	Severe

#### TGF-β1 and IL-6, and levels measurement

According to [31], the mRNA levels of TGF-β1 and IL-6 were measured 30 days postoperatively in the five rats. It was decided to gather the scar tissue from the laminectomy locations. Trizol reagent was used to extract the total RNA, and a Promega reverse transcriptase kit was used to convert the RNA (2 μg) into cDNA. Following the methods outlined in prior work, the Bio-Rad MYIQ2 (USA) apparatus was used to perform the triple quantitative procedure known as qRT-PCR. Table 2 lists the primer sequences for the genes under investigation. As an internal control, GAPDH amplification was used.

**Table 2:** The primer sequences of IL-6 and TGF-β1 genes

Gene	Product size	Sequence
TGF-β1	101	Reverse: CACTGCTTCCCGAATGCTGA Forward: TGCTAATGGTGGACCGCAA
IL-6	105	Reverse: GTTTGCCGAGTAGACCTCATAG Forward: GAAGTTAGAGTCACAGAAGGAGTG
GAPDH	187	Reverse: AGTTGAGGTCAATGAAGGGG Forward: CTCTGCTCCTCCCTGTTCTA

### Statistical analysis

The results were analyzed using the 22nd edition of SPSS software (IBM, USA). The Shapiro test was used to confirm that the data were normal. Means ± SE were used to express quantitative data. A one-way ANOVA test was used, and to compare the different groups, Tukey’s post hoc test was performed. A *P*-value < 0.05 indicated statistical significance. Kruskal–Wallis, followed by Dunn’s test, was used to statistically analyze the severity of inflammation. Different small alphabetical letters referred to significance when *P* < 0.05. The Chi-square test (Pearson and exact Fisher) was used to analyze the significance of the relationship between the various groups and the qualitative data (EF and epidural scar adhesion scores).

## Results

### Macroscopic evaluation of epidural scar adhesion

Severe epidural adhesions were found in the laminectomy group, with nine rats (90%) graded as grade 3 and one rat (10%) as grade 2. These results were significantly different (*P* < 0.002) compared to the other groups. The Fisher values between the laminectomy group and the control, PRF, chitosan, and combination PRF/chitosan groups were 21.8, 12.3, 13.4, and 21.5, respectively.

Moderate-to-severe grades of epidural adhesions were observed in the PRF and chitosan-treated groups, with no significant differences noted (*P* 0.9 and Fisher’s value 1.5). The epidural adhesion grade was 0 in two rats (20%) in both groups, while grade 1 was observed in five rats (50%) in the PRF group versus three rats (30%) in the chitosan group. Besides, two rats (20%) in the PRF group and four rats (40%) in the chitosan group were classified as grade 2. Only one rat (10%) in each group was classified as grade 3.

**Fig. 4:**
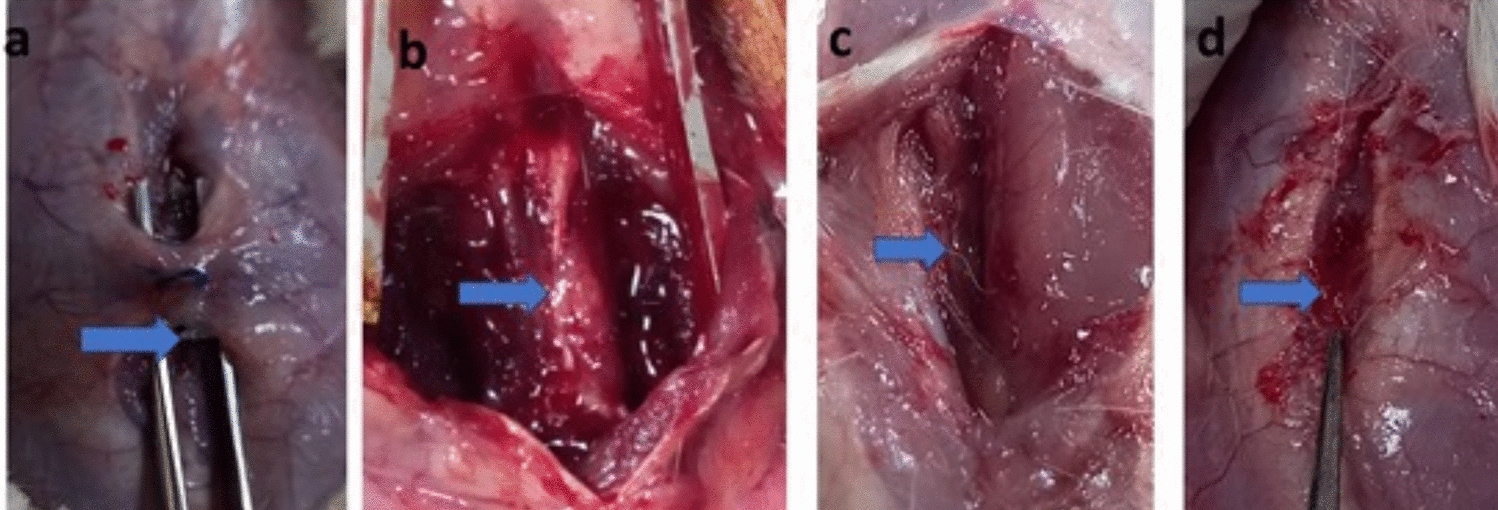
Macroscopic assessment of epidural scar adhesion grades among groups. **A** The control one showed a grade of zero. **B** The laminectomy group showed a grade of three. **C** The PRF and chitosan groups showed a grade of two. **D** The combination PRF/chitosan group showed a grade of one

On the other hand, the PRF/chitosan group achieved a significantly milder degree of epidural adhesions post-surgery (*P* < 0.05) than other groups. Nine rats (90%) achieved an epidural adhesion grade of 0, while only one rat (10%) was classified as grade 1 (Fig. 4).

### Histological analysis

#### By hematoxylin and eosin (H&E) stain

**Fig. 5:**
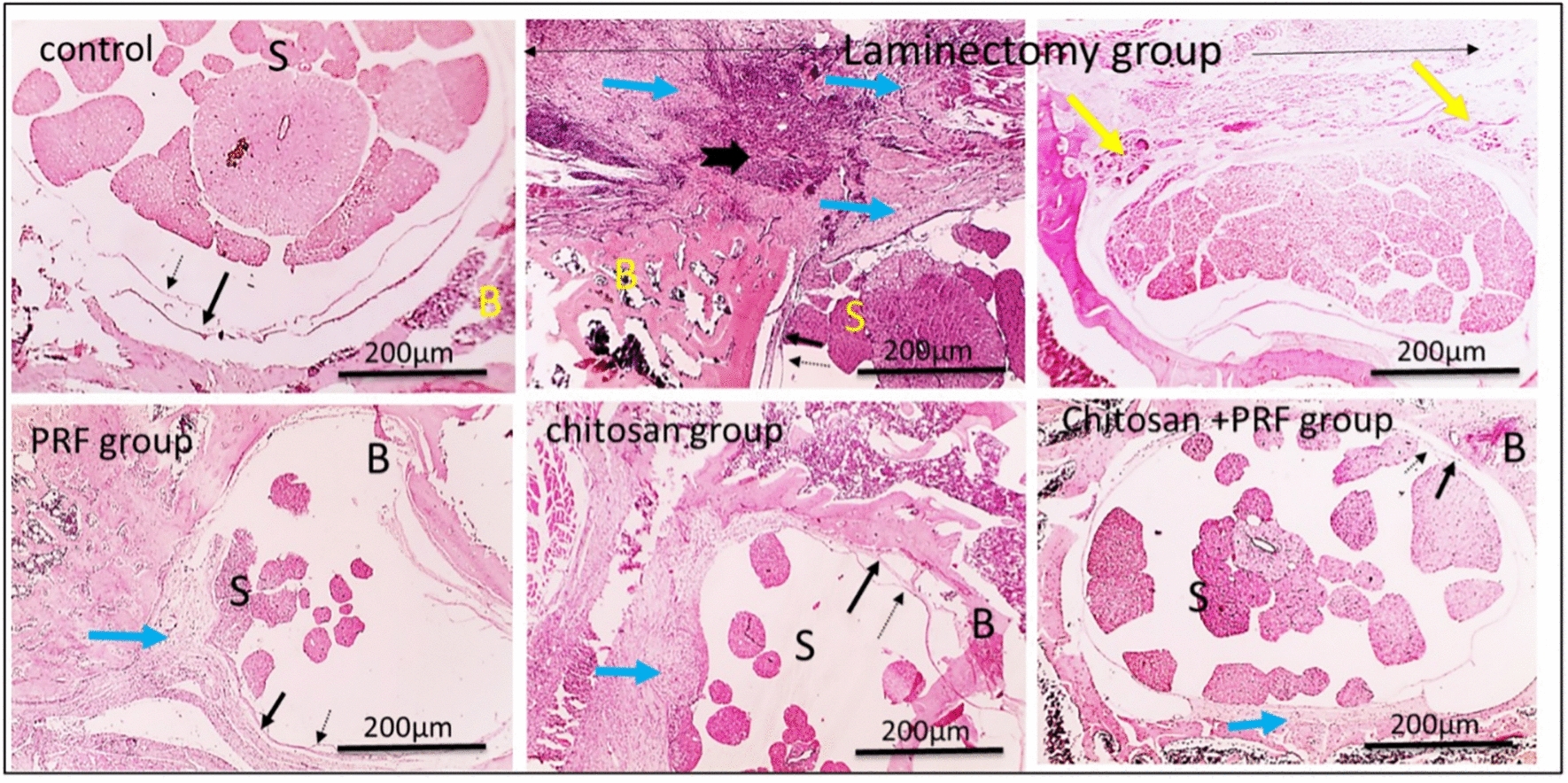
Histopathological evaluation by H&E stain; X: 40 bar 200μm and X: 100 bar 100μm. The control group contains normal dura mater (black arrow), pia mater (dashed black arrow), bone (B), and spinal cord (S) components. The laminectomy group revealed an extensive epidural deposition of scar tissue (blue arrow), a granuloma formation (yellow arrow), and severe inflammation (thick black arrow) surrounding the spinal cord, which led to adhesion to the pia mater and increased dura mater thickness. Inflammation and the formation of epidural scar connective tissue showed a significant reduction in the PRF group (blue arrow). The chitosan group exhibited moderate inflammation and the production of epidural scar tissue (blue arrow), which resulted in increased dura mater thickness and partial adherence to the pia mater. The combined group showed mild epidural fibrosis (blue arrow)

The control normal group showed a normal anatomical structure of the vertebrae, dura mater, arachnoid, and spinal cord (Fig. 5); whereas, the laminectomy group had massive inflammation, dystrophic calcification, granuloma development, and excessive epidural scar tissue deposition found around the spinal cord, resulting in thicker dura mater and visible adhesion with the pia mater. Additionally, in vertebral sections from the laminectomy group, there was extensive leukocytic cell infiltration, dystrophic calcification, granuloma formation, and excessive scar tissue deposition with or without adherence to the dura mater (Fig. 5). The treated groups showed a significant decrease in inflammation scores. In the chitosan group, there is partial adherence to the pia mater and increased dura mater thickness due to moderate inflammation and the development of scar tissue around the spinal cord. Additionally, there is noticeably reduced inflammation and the formation of epidural scar tissue around the spinal cord in the PRF group, while in the combined group, mild epidural fibrosis is observed (Fig. 5).

#### Masson trichrome (MT) stain

**Fig. 6:**
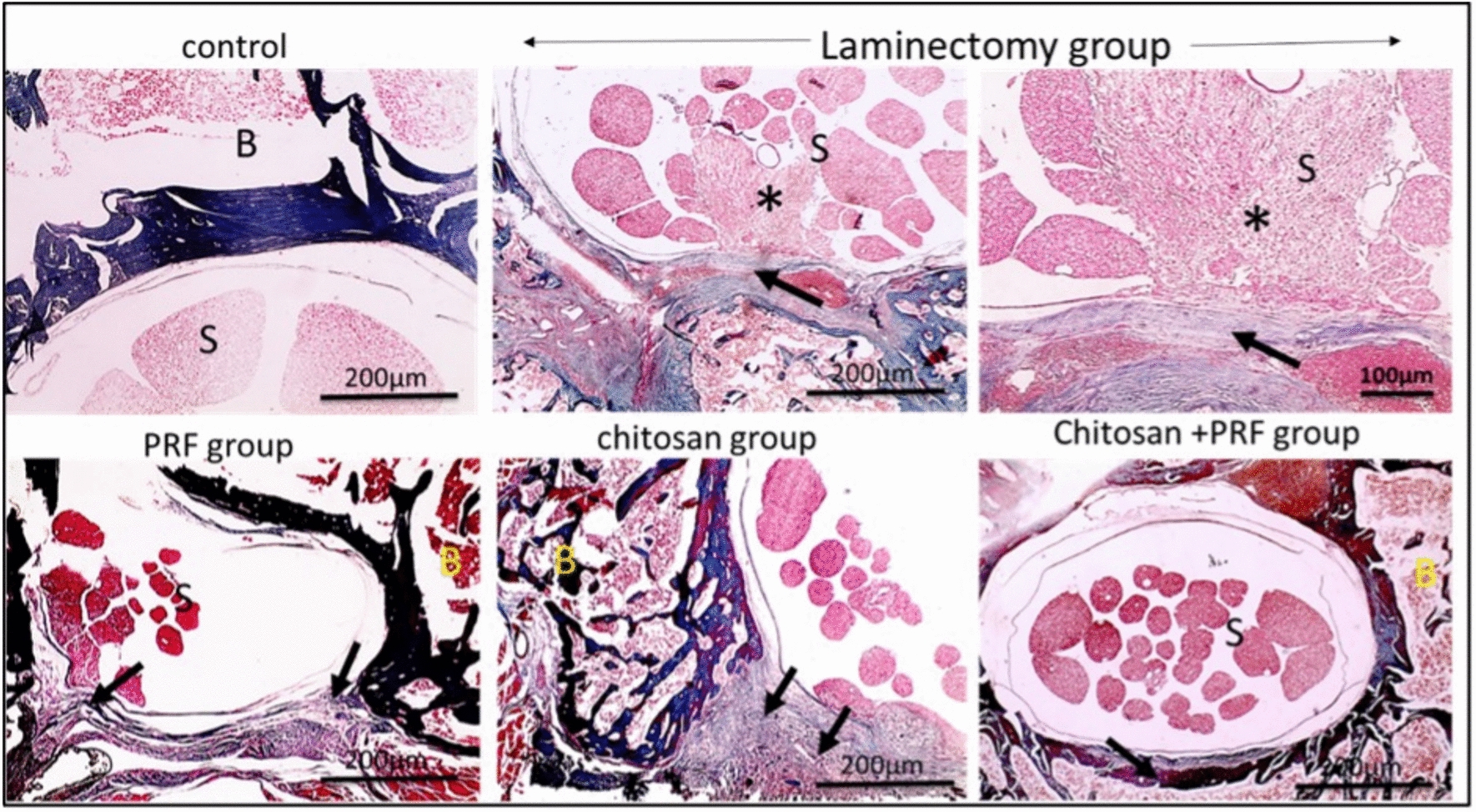
Microscopic pictures, MT stain; X: 40 bar 200μm. The control group does not exhibit fibrous tissue deposition. The laminectomy group exhibits excessive fibrous tissue epidural deposition (black arrow), granuloma formation (yellow arrow) surrounding the spinal cord, and thicker dura mater invasion of nervous tissue (*) in certain areas. The PRF group has significantly less epidural fibrous connective tissue formation (black arrow). The chitosan group exhibits a substantial quantity of epidural scar connective tissue (black arrow). The PRF/chitosan group, mild epidural fibrosis (black arrow) is observed. S: spinal cord, B: bone

**Fig. 7:**
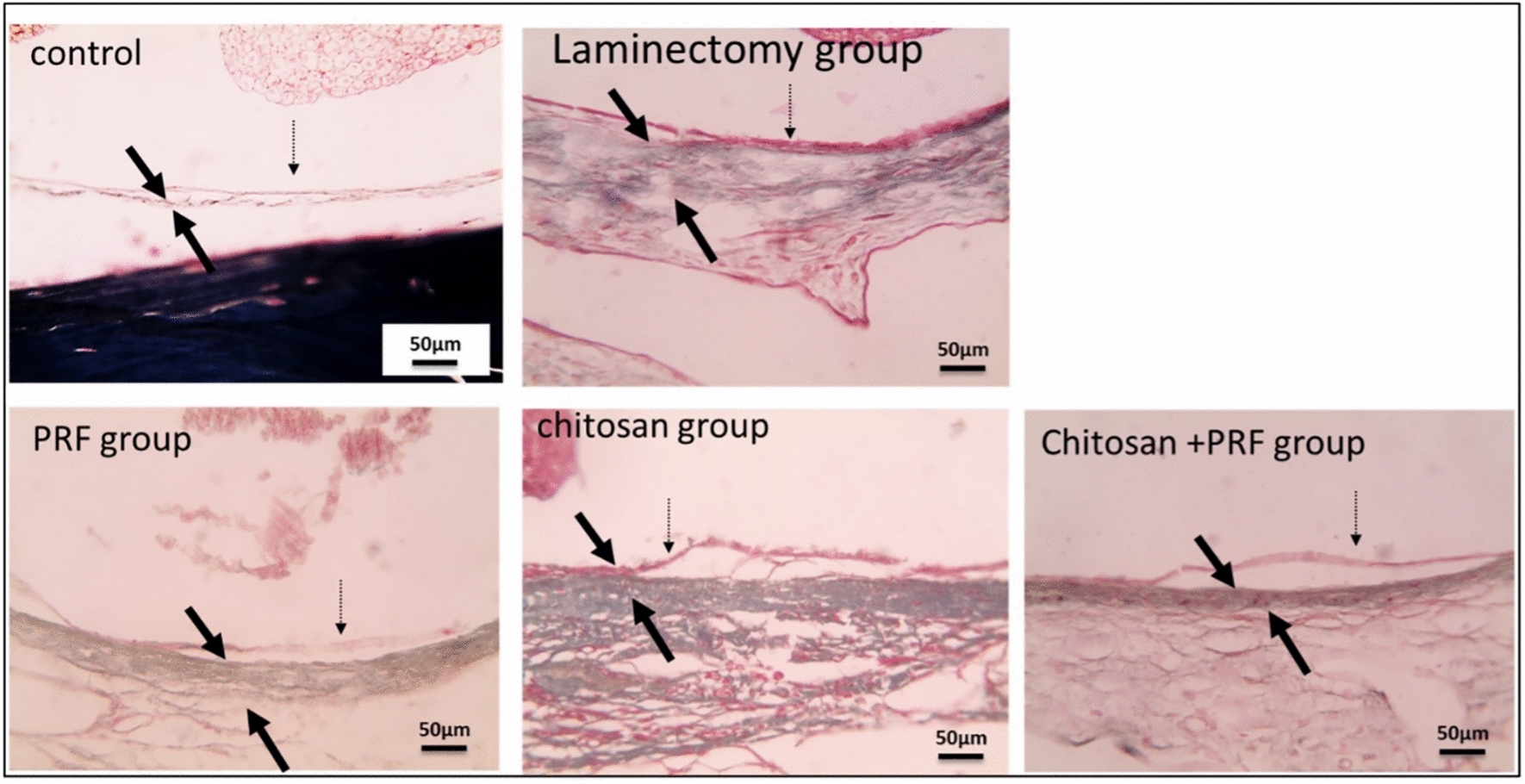
Histopathological analysis by MT stain; X: 400 bar 50 μm. Dura mater (thin black arrow); pia mater (dashed black arrow). The control, PRF, chitosan, and PRF/chitosan groups showed a reduction in the thickness of the dura mater compared to the laminectomy group

In the control group, no fibrous tissue deposition was seen. However, excessive fibrous tissue deposition, formation of granuloma around the spinal cord, and thicker dura mater in some places that invaded nerve tissue were all observed in the laminectomy group. All treated groups showed reduced scar tissue and epidural thickness as compared to the laminectomy group. The chitosan group exhibited a substantial degree of scar tissue surrounding the spinal cord; the PRF exhibited significantly less development of epidural fibrous tissue surrounding the spinal cord, and the combined group had mild epidural fibrosis (Fig. 6). Moreover, the higher magnification X:400 MT stain showed that all treatment groups' dura mater thickness was lower than that of the laminectomy group (Fig. 7).

#### Histological grading score of inflammation, dura mater thickness, and density of the scar

**Fig. 8:**
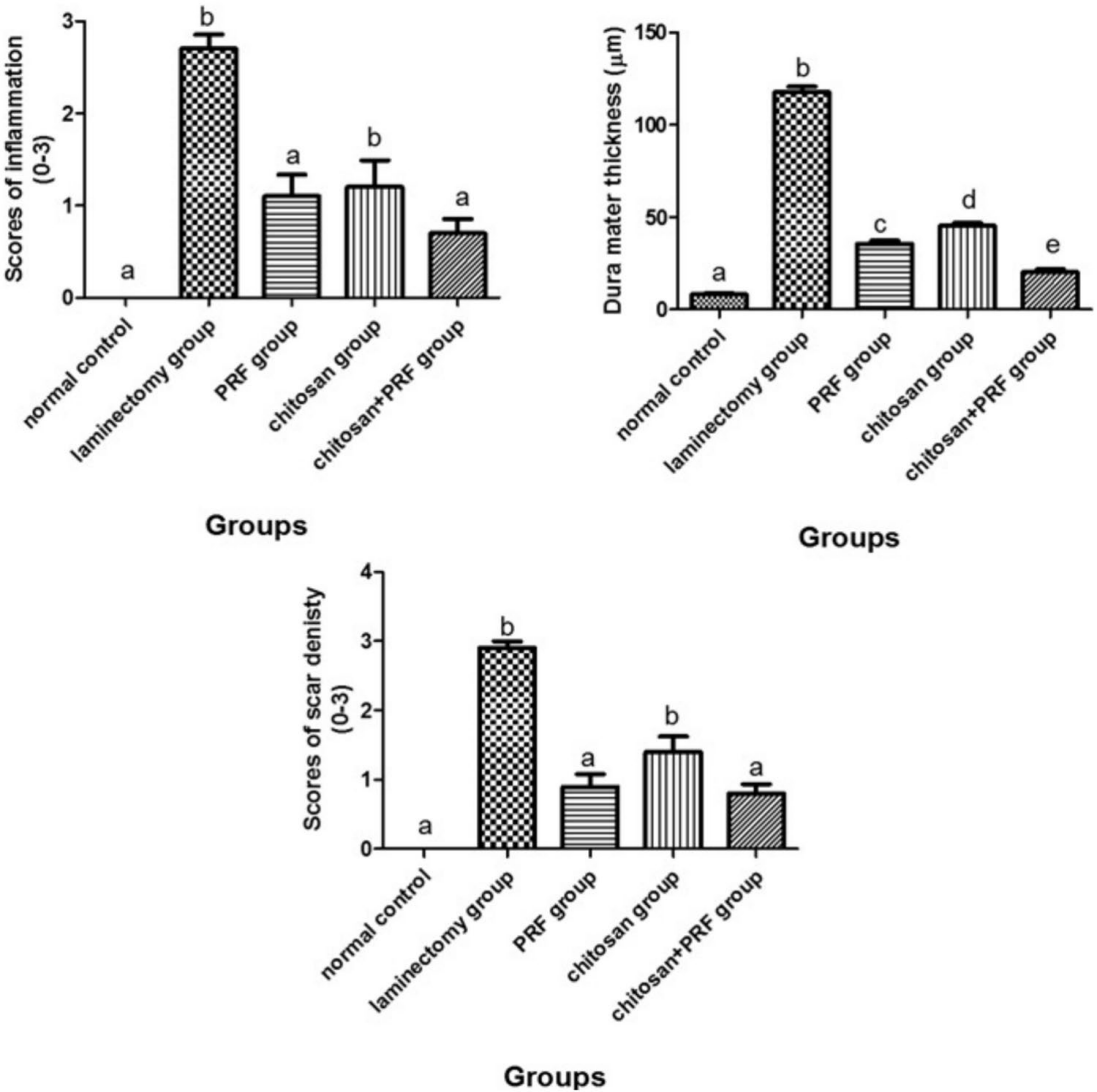
Inflammation scores significantly decreased in the treatment groups (PRF, chitosan, and combined) compared to the laminectomy group. Scare tissue density score also showed a significant decrease in the treatment groups compared to the laminectomy group. In addition, Dura mater thickness significantly decreased in all the treated groups compared to the laminectomy group. Different small alphabetical letters indicate significance when the *P* value < 0.05

Compared to the laminectomy group, all treatment groups showed a significant decrease (*P* < 0.001) in inflammatory scores as well as a significant reduction in dura mater thickness and the density of the scar. The scores were severe (3) in the laminectomy group, moderate (2) in both the PRF and chitosan groups, mild (1) in the combined group, and no inflammation score (0) in the control group (Fig. 8). Arachnoiditis was detected post-surgery in all groups: 10 rats in the laminectomy group, 5 rats in the PRF group, 7 rats in the chitosan group, and 4 rats in the combined group, while the control group was unaffected. The arachnoid layer was inflamed and involved in all rats in the laminectomy group. In contrast, seven rats were recorded in the chitosan group, five rats were recorded in the PRF group, and four rats were recorded in the PRF + Chitosan group.

### The level of TGF-β1 and IL-6 genes

**Fig. 9:**
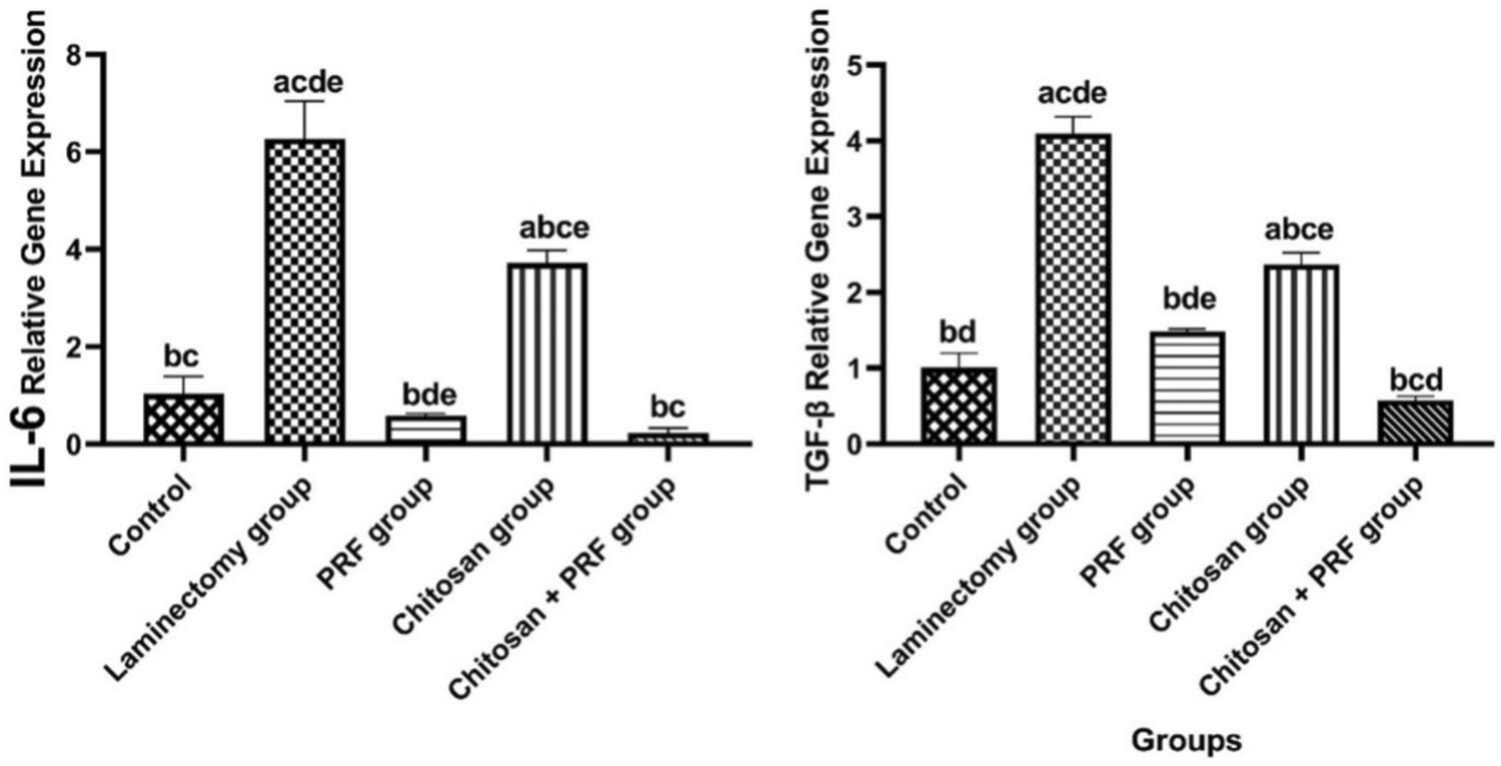
The degree of TGF-β1 and IL-6 mRNA expression in each group’s epidural scar tissue. The relative mRNA expression level was assessed using Kruskal–Wallis statistical analysis and Dunn’s test RT-PCR testing in comparison to the laminectomy group. The IL-6 and TGF-β1 expression level in the combined group was more significantly decreased (*P* < 0.0001) compared to the other groups, data are presented as mean ± SD. Different superscript letters indicate significant change among groups

The IL-6 level in the laminectomy group was significantly increased compared to the control group. The level of IL-6 expression was significantly lower in the PRF group than in the laminectomy group. In contrast to the laminectomy group, its level was significantly downregulated in the chitosan group (*P* < 0.0005). When compared to the laminectomy group and other treatment groups, the combined group’s IL-6 expression level dropped dramatically (*P* < 0.0001; Fig. 9).

TGF-β1 fibrosis marker level was markedly elevated (*P* < 0.0001) in the laminectomy group than in the control group. In the PRF group, the TGF-β1 expression level is significantly decreased (*P* < 0.0001) than the laminectomy group. The TGF-β1 expression level was markedly decreased in the chitosan group compared to the laminectomy group. However, its level was higher than the group PRF& chitosan group (*P* < 0.0001). The TGF-β1 expression level in the combined group significantly decreased compared to the laminectomy group and all other treated groups (*P* < 0.0001: Fig. 9).

## Discussion

In the laminectomy procedures for disc herniation and stenosis, EF is a significant contributor to FBSS. EF may result in radicular symptoms that resemble surgical symptoms due to dural entrapment or radix compression. This exacerbates discomfort and suffering [32] and significantly reduces the success probability of repeat surgery to 5–30% [33]. Additionally, repeated procedures increase EF rates, which results in ongoing suffering for patients. The purpose of this study was to eliminate EF in a rat model by combining PRF and chitosan, since EF reduction is necessary after laminectomy.

The main process in the creation of EF is the fibroblasts’ rapid proliferation brought on by the activation of growth factors and cytokines. When a laminectomy is performed, this proliferation attempts to repair the locally injured spinal region [34].

EF is a consequence of the body’s natural wound-healing mechanism. Because EF causes adhesions and scar tissue in the healing area, patients who have recurrent surgery are more prone to experience complications like nerve root injury, dura damage, epidural hematoma, and infection. As a result, research into preventing the creation of EFs has been increasingly important in recent years [2, 7, 14, 15, 35–38]. In our study, male Sprague Dawley rats were used for EF formation following lumbar laminectomy in rats that may be affected by endogenous estrogen [7].

The macroscopic examination of epidural scar adhesion in a laminectomy group demonstrated substantial progression to higher grades, with 40% of participants classified as Grade 2 and 60% as Grade 3. This result is a result of fibroblasts’ rapid proliferation and the cytokine and growth factor activation this result agrees with [34].

The natural wound-healing process causes epidural scar adhesion, as shown by the Masson trichrome stain. Around the spinal cord, there is dystrophic calcification, granuloma development, and severe inflammation, which results in a thicker dura mater and noticeable adhesion to the Pia mater. These results agree with [2, 36, 39]. Furthermore, the laminectomy group shows the highest mean expression of IL6 and TGFβ-1 in laminectomy rats than in the control group and other treated groups due to epidural fibrosis formation, which agrees with the prior studies [40, 41].

After laminectomy, the efficacy of PRF and chitosan in eliminating the EF was evaluated; in contrast to the laminectomy group, PRF reduced inflammation and the formation of epidural scar tissue surrounding the spinal cord. Improved osteoblast proliferation, angiogenesis, reduced epidural hemorrhage, and cytokine-assisted tissue regeneration are all responsible for the reported outcomes [2, 42]. Platelet-derived growth factor indicated that fibrin-containing gels enhanced EF in the first 2 weeks after laminectomy and that platelet concentrate-containing growth factor reduced fibrotic tissue by promoting the reperfusion of injured muscle tissue [43]. Additionally, compared to the laminectomy group, PRF-treated rats showed a significant elimination of TGFβ-1 and IL6. This was carried out to verify the histopathological scoring associated with the previously mentioned benefits of PRF.

According to the main finding, PRF may enhance wound healing and soft tissue regeneration. Furthermore, PRF is a safe, dependable, and cost-effective solution for accelerating wound healing and improving the efficiency of tissue repair after damage or injury [44]. The results of our study showed that in the PRF-treated group, 20% of rats had grade 0 epidural scar adhesion according to Rydell’s classification, 40% had grade 1, 30% had grade 2, and 10% had grade 3. The aforementioned results mean that PRF can reduce epidural scar adhesion than the laminectomy group. This result confirms previous findings [2] that PRF eliminates epidural adhesion by alleviating fibroblast proliferation and inflammatory cell infiltration.

The PRF group underwent quantitative morphometric analysis to determine the grading of EF. The results revealed that 30% of rats had grade 0.50% had grade 1.10% had grade 2, and 10% had grade 3. This led to a substantial reduction in comparison to the laminectomy group [2, 45]. These findings support the notion that the locally approved PRF is a highly efficient, secure, and uncomplicated method for reducing EF in patients who have undergone laminectomy. The histological findings indicate that arachnoidian involvement was observed in only 50% of rats, representing a significant decrease compared to the laminectomy group, which agrees with [4, 46].

Statistical analysis of scar tissue density scores and dura mater thickness showed a marked decline in the treated PRF group compared to the laminectomy group, which results agree with [2].

Chitosan, a natural polymer derived from chitin deacetylation (poly-*N*-acetylglucosamine), has been widely employed in topical dressings in wound healing due to its stypticity, antibacterial and nontoxic, biocompatible, and biodegradable qualities [47–49]. It has been demonstrated that chitosan inhibits collagen production and fibroblast growth. Chitosan acts as an effective barrier for collagen penetration that prevents the spinal cord from EF after a disc injury [50]. Recently, Javdani et al. [51] found that chitosan hydrogel has the ability to reduce inflammation and oxidative stress markers such as catalase, glutathione peroxidase (GPx), superoxide dismutase (SOD), and malondialdehyde (MDA) following traumatic spinal cord injury (SCI) in rats. Additionally, Shamsi et al. [52] reported that in diabetic rats, chitosan enhances skin wound healing and angiogenesis by stimulating the infiltration of inflammatory cells.

The selected volume of 1.25 mL of acetic acid in 50 mL of deionized water (2.5% v/v) was chosen based on a balance between ensuring efficient solubilization of chitosan and maintaining mild acidic conditions to preserve the functional properties of the resulting solution. Chitosan is known to be insoluble in neutral water, but readily dissolves in dilute acidic media due to the protonation of amino groups (–NH₂) on the chitosan backbone. A concentration around 1–2% v/v acetic acid is commonly reported in the literature as effective for dissolving chitosan with a degree of deacetylation higher than 80%. The selected 2.5% v/v acetic acid ensures complete protonation of chitosan without causing excessive acidity that could degrade the polymer chains or interfere with downstream interactions. Furthermore, this concentration provided a clear, homogeneous solution within 2 h of stirring at 500 rpm, indicating sufficient dissolution. Using significantly higher concentrations of acetic acid is unnecessary and may lead to excessive acidity, which could affect the stability or reactivity of both chitosan, and any additives or fillers used in subsequent processing. Therefore, the 1.25 mL in 50 mL ratio was selected as a standardized and efficient condition to ensure complete dissolution of chitosan while maintaining the desired physicochemical stability of the solution [28].

In the current study, the macroscopic examination of the Chi group exhibited a diverse distribution, with participants across all grades (10% in Grade 0, 20% in Grade 1, 40% in Grade 2, and 30% in Grade 3) because of its nontoxic, antibacterial, biocompatible, and biodegradable qualities [53] with fibroblast growth and collagen synthesis inhibition [50].

By Masson trichrome stain, moderate inflammation, and epidural scar tissue formation were seen around the spinal cord, and partial adhesion to the pia mater; these results are due to chitosan significantly inhibiting the deposition of collagen at the site of injury and having a modulatory-inhibitory impact on fibroblasts, as mentioned by [54, 55]. On the other hand, the chitosan group does not show significant differences in IL6 and TGFβ-1 expression when compared to the control group. This indicates that these treatments may not have a substantial effect on their levels. This result disagrees with [56] who mentioned that chitosan promotes wound closure by inducing TGFβ1, FGF, PDGF, and ECM deposition.

One of the limitations of this study was that it only evaluated dependent macroscopic and microscopic variables, without analyzing biomechanical or quantitative analyses. Additionally, only one time point was selected to assess the levels of epidural fibrosis and adhesion. Furthermore, there was a lack of diagnostic imaging techniques necessary to monitor progress during and after the surgery.

## Conclusion

Chitosan with PRP has beneficial effects on limiting EF formation after laminectomy in rats compared to using chitosan or PRF alone. It could be considered a promising therapy following spinal surgery to reduce the risk of EF complications.

## Data Availability

Data is provided within the manuscript.

## Abbreviations

EF: Epidural fibrosis
FBSS: Failed back surgery syndrome.
Chi: Chitosan
PRF: Platelet-rich fibrin
L3–L5: Lumber vertebrae 3–5
IL-6: Interleukin 6
TGF-β1: Transforming growth factor beta
